# Glycemic-aware metrics and oversampling techniques for predicting blood glucose levels using machine learning

**DOI:** 10.1371/journal.pone.0225613

**Published:** 2019-12-02

**Authors:** Michael Mayo, Lynne Chepulis, Ryan G. Paul

**Affiliations:** 1 Department of Computer Science, University of Waikato, Hamilton, New Zealand; 2 Waikato Medical Research Center, University of Waikato, Hamilton, New Zealand; 3 Waikato Regional Diabetes Service, University of Waikato, Hamilton, New Zealand; Wroclaw University of Science and Technology, POLAND

## Abstract

Techniques using machine learning for short term blood glucose level prediction in patients with Type 1 Diabetes are investigated. This problem is significant for the development of effective artificial pancreas technology so accurate alerts (e.g. hypoglycemia alarms) and other forecasts can be generated. It is shown that two factors must be considered when selecting the best machine learning technique for blood glucose level regression: (i) the regression model performance metrics being used to select the model, and (ii) the preprocessing techniques required to account for the imbalanced time spent by patients in different portions of the glycemic range. Using standard benchmark data, it is demonstrated that different regression model/preprocessing technique combinations exhibit different accuracies depending on the glycemic subrange under consideration. Therefore technique selection depends on the type of alert required. Specific findings are that a linear Support Vector Regression-based model, trained with normal as well as polynomial features, is best for blood glucose level forecasting in the normal and hyperglycemic ranges while a Multilayer Perceptron trained on oversampled data is ideal for predictions in the hypoglycemic range.

## Introduction

Type 1 Diabetes (T1D) is an autoimmune disease where the pancreas produces little to no insulin [1]. Conventional therapy requires patients to inject themselves with insulin multiple times per day. However, with more recent advancements in technology, specifically systems known as artificial pancreases (APs), improved glycemic control is now possible [2].

The standard AP consists of three main components. Firstly, there is a continuous glucose monitor (CGM) which monitors glycemic levels via a small sensor inserted subcutaneously in either the forearm or the abdomen. The second component is an insulin delivery system, typically a continuous pump, which delivers insulin at either a user-specified or an automatically determined basal rate, also subcutaneously. Thirdly, there is a micro-controller linking the two devices together wirelessly, whose main purpose is regulating the insulin pump rate such that time spent in normoglycemia is maximised. Additionally, a dedicated reader or a smartphone may also be used so the patient can observe their current and recent past glucose levels, along with the status of the pump, in real time or on-demand.

Despite these technological improvements, most patients with T1D are still not able to achieve near-normal glycemia, and remain at risk of severe hypoglycemia and diabetic ketoacidosis [3, 4], albeit with a lower probability than patients undergoing non-AP treatment. This risk is primarily due to the insulin pumps themselves not responding adequately to changing glycemia, one dimension of which is the inability of the controller to accurately forecast short term future glycemic levels.

Consequently, several studies have reported on the benefits of being able to predict hypoglycemia in patients with T1D as a way to improve clinical outcomes [5–7], and alerts can easily be built into existing monitoring software if the patient is using a CGM only or a full AP [8, 9]. In this paper, the focus is on the application of machine learning for predicting glycemic levels which can then be used for generating alerts. Examples of the current level of interest in applying artificial intelligence and machine learning techniques to this problem are illustrated by two recent extensive survey papers on the topic [10, 11].

The main contributions of this paper are as follows. Firstly, the predictive performance metrics used for assessing different machine learning techniques and selecting the best single technique are considered. It is shown, via an extensive set of experiments, that the best metric should focus on the portion of the range where the highest accuracy is desired. For example, if the ultimate aim is to generate alerts whenever a patient is at risk of hypoglycemia (<70 mg/dl blood glucose level), then the chosen metrics should focus on this range in addition to the overall range. In contrast, recent works in the literature (e.g. [12] as well as those covered in the survey articles [10, 11]) usually compute accuracy over the entire glycemic range, which may lead to misleading conclusions. A solution to this problem is to break the glycemic range into meaningful parts and then analyse each subrange separately.

Secondly, once the importance of this approach is established, further analysis in this paper then shows that the naive application of machine learning models to the data does indeed lead to bias. This is because most T1D patients spend most of the time in the normal range. Since there is very little work in the literature on the problem of imbalanced regression [13], an adaptation of standard preprocessing techniques for imbalanced labelled data, applied to blood glucose level regression, is proposed here. This in turn leads to demonstrated increases in accuracy on particular subranges of the glycemic range.

## Materials

The data analysed is reviewed in this section and then the machine learning and preprocessing methods used are summarised. Finally, typical metrics for assessing blood glucose level predictors from the literature are described.

### Ohio T1D dataset and prediction task

The University of Ohio T1D Dataset [14] is a publicly available benchmark dataset comprising data from six T1D AP users taken over a period of approximately eight weeks. The dataset is nontrivial in size and consists of detailed information from the patients including interstitial glucose levels recorded by a Medtronic Enlite CGM, records of doses of basal and prandial insulin administered by a Medtronic 530G insulin pump (including temporary basal rate changes and corrective boluses), physiological data recorded by a Basis Peak fitness band (e.g. heart rate) and a variety of self-reported data (such as self-reported sleeping times and food intake estimates). A period of approximately eight weeks is covered for each patient which in turn is split chronologically into two parts: approximately the first six weeks of data are reserved for training, and approximately the last two weeks are reserved for testing.

**Fig 1:**
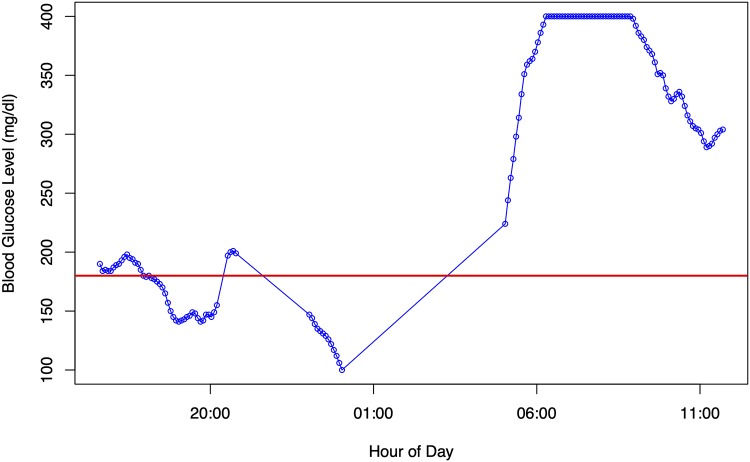
A sample of a few hours of CGM data from one patient. The horizontal red line indicates the boundry between normoglycemia and hyperglycemia according to [15]. Note (i) the two gaps in the trace, one shorter and one longer; and (ii) maximum possible sensor reading of 400 mg/dl, even though glucose levels can exceed this amount. This patient experienced hypoglycemia just after 1am followed by severe hyperglycemia later in the morning.

In this paper, the focus of analysis is on the timestamped CGM portion of the data. An example of this type of data is given in Fig 1 which shows a fragment of CGM trace for one patient over a period of approximately twenty hours. The horizontal lines on the graph are at 70 mg/dl and 180 mg/dl respectively which are are the thresholds for level 1 hypoglycemia and level 1 hyperglycemia [15]. The normoglycemic range lies between these ranges.

A typical issue when dealing with this type of CGM data is gaps in the CGM trace which may occur frequently and for multiple reasons. For example, sensor replacement necessarily leads to a gap in the data of a varying amount of time; likewise forgetting to swipe a flash-type sensor within eight hours may also lead to a gap. Both types of data gap are understandable and expected.

However, dealing with these gaps can be problematic from a machine learning point of view. Currently, it is not clear how to impute missing CGM trace data. Previous works have addressed the problem of gaps by applying time series imputation methods, e.g. Kalman Smoothing [12], but such methods may lead to bias since they require information from the future before the imputation can be performed. Similarly, simple methods such as “last value carried forward” may also induce bias.

In this work, the missing value problem is circumvented by considering only complete trace fragments. To construct examples for the training and testing data, a sliding prediction window technique is used. The prediction window length is 120 minutes (equivalent to 24 consecutive CGM readings at an interval of five minutes between readings) with the target value for each prediction window being CGM reading 30 minutes after the end of the prediction window. If a sliding prediction window has less than 24 readings, it must contain a gap and therefore it is discarded. Table 1 gives the number of examples in the training and testing datasets for each patient after this process. The table also shows the patient identifiers from the Ohio T1D dataset as well as the randomly-shifted date ranges that each dataset covers to emphasise that the test data occurs after the training data.

**Table 1:** Number of examples in each training set after processing the CGM traces with the sliding window technique, along with the start and end dates (randomly shifted) for each dataset.

Patient	Training Size	Train Dates	Testing Size	Testing Dates
559	9,517	7/12/21–17/1/22	2,163	18/1/22–27/1/22
563	11,500	13/09/21–28/10/21	2,460	29/10/21–7/11/21
570	10,364	7/12/21–16/1/22	2,453	17/1/22–26/1/21
575	9,821	17/11/21–1/1/22	2,289	2/1/22–11/1/21
588	12,332	30/8/21–14/10/21	2,701	15/10/21–24/10/21
591	10,088	30/11/21–13/1/22	2,621	14/1/22–23/1/22

**Fig 2:**
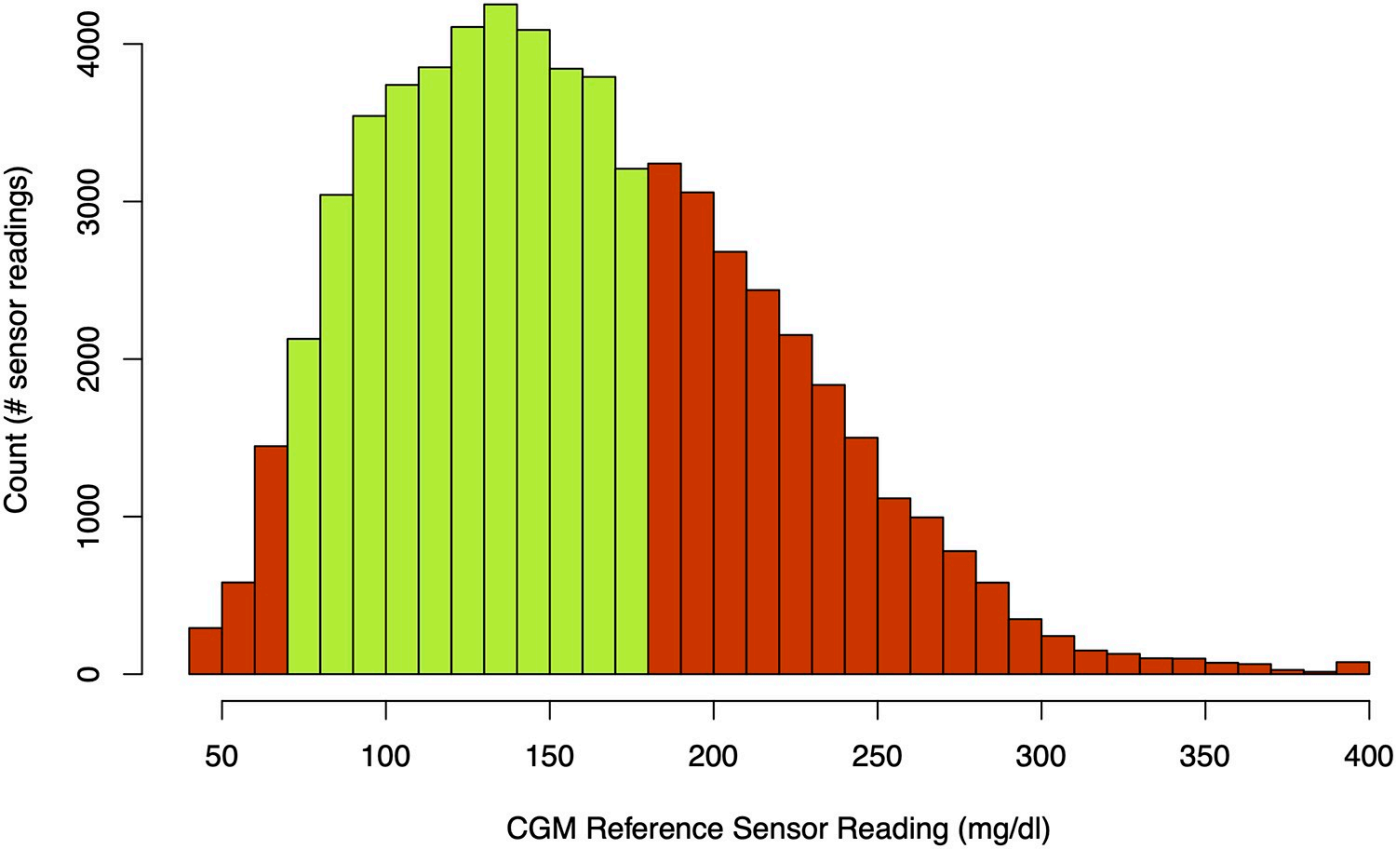
Frequency histogram showing counts of CGM sensor readings for all patients in the training data. Different colours indicate whether or not the sensor reading is normoglycemic or not.

In the Introduction, it was mentioned that the CGM data is imbalanced across the different glycemic ranges because T1D patients spend most of their time in normoglycemia. This is illustrated in Fig 2, which depicts a frequency histogram over CGM sensor readings across the entire Ohio dataset. The figure clearly shows that the bulk of the sensor readings are normoglycemic with only 2,141/63,622 (3.4%) being hypoglycemic and 21,707/63,622 (34.1%) being hyperglycemic. Therefore imbalance is a serious issue in this data, and consequently it can be expected that most models learned using machine learning algorithms will be biased towards predicting values in the normoglycemic range.

### Machine learning regression models

In this section, ten different machine learning algorithms used for training regression models are outlined. These models are well known in the literature, and where appropriate citations are provided. The models are described in approximate order from the most interpretable (i.e. so-called “transparent” or “white box” models) to the least interpretable (so-called “black box” models).

#### Dummy

The dummy model constantly predicts the mean blood glucose level of the patient, computed from that patient’s training data. As such, it is not a “true” model and poor predictive performance is expected, but it is included as a baseline method for comparison with the other approaches.

#### Lasso

The Lasso algorithm constructs a sparse linear predictive model [16, 17]. The model is sparse in that it uses L1 regularisation to set as many model coefficients to zero as possible. It is well known that blood glucose dynamics are distinctly non-linear and therefore Lasso in its naive direct application would not be suitable for this problem. The Lasso algorithm is therefore modified to enable it to capture non-linear dynamics by creating additional “polynomial” features from the original features, and training the model on this expanded set of features. Eq 1 illustrates this, with ${\hat{bg}}_{t+30}$ being the model’s thirty minutes ahead prediction, *x*_*i*_ being the *i*th CGM sensor reading in the sliding prediction window, and *β*_*i*_ being the *i*th coefficient of the model.

$$\begin{array}{c} \begin{array}{cc} {\hat{bg}}_{t+30} & =\beta_{0}+\beta_{1}x_{1}+\beta_{2}x_{2}+\cdots +\beta_{24}x_{24}\\ & +\beta_{25}x_{1}^{2}+\beta_{26}x_{1}x_{2}+\cdots +\beta_{48}x_{1}x_{25}\\ & +\beta_{49}x_{2}^{2}+\beta_{50}x_{2}x_{3}+\cdots \\ & \cdots \end{array} \end{array}$$ (1)

Eq 1 shows that the first twenty five terms correspond to a linear model consisting of an intercept and one coefficient per input variable. However, subsequent terms correspond to non-linear inputs which are either squares of each individual input or interactions calculated by taking feature products. While a Lasso model trained on normal and polynomial features has a significantly higher dimensionality than a model trained using normal features only, the tendency towards sparsity induced by L1 regularisation should produce a model that is both accurate and concise.

#### Support vector regression (linear kernel)

An alternative method for training an additive linear model on a set of features and their polynomial combinations is to use the support vector regression (SVR) [18] instead. Support vector machines were originally formulated for binary class (i.e. positive vs. negative) classification. The basic idea is to find via an optimisation process the so-called “maximum margin hyperplane” that separates examples from the two classes with the widest possible margin. This idea can also be applied to regression, but instead of maximally separating examples, the hyperplane that best fits the target values of the examples is found instead. Predictions are made by evaluating this hyperplane at new points. In this approach, L1 regularisation is still utilised so that as many polynomial features are eliminated as possible. SVR-based linear models often produce significantly more accurate models than other linear regression approaches such as Lasso.

#### Decision tree

**Fig 3:**
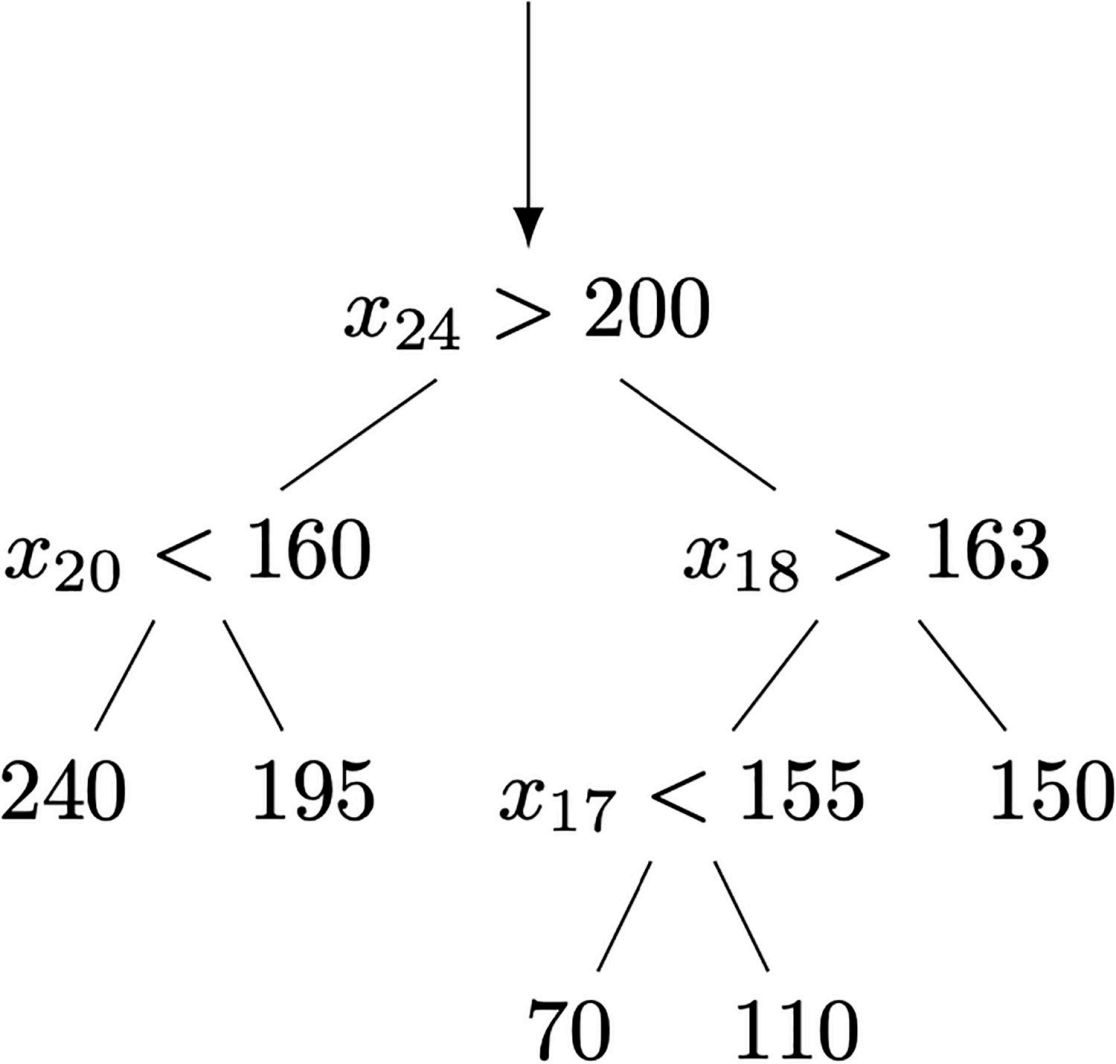
Illustration of a decision tree used for regression. Intermediate nodes represent tests of the features and leaf nodes are predictions for ${\hat{bg}}_{t+30}$.

A decision tree is a non-parametric approach to learning a regression model [19]. The basic idea is to repeatedly learn simple decision rules, each rule focusing on a single basic feature in the data. For example, if *x*_3_ represents the CGM reading twenty minutes ago in a prediction window, then a simple decision rule involving this feature is “if *x*_3_ > 140 mg/dl then predict 160 mg/dl else predict 150 mg/dl”. Multiple decision rules can be stacked on each other, producing a decision tree. Fig 3 illustrates a very simple decision tree. Advantages of decision trees include simplicity, ease of understanding, and the relative straightforwardness of algorithms for learning such a tree from data. On the other hand, decision tree learning algorithms may produce models that are overly complex and/or biased if the data is imbalanced.

#### K-Nearest Neighbour

Like decision tree learning algorithms, the K-Nearest Neighbour algorithm is relatively simple. The idea is that instead of building a model explicitly, the entire training dataset is simply memorised. When a prediction for a new example is required, the K closest examples to the new example are determined using a particular distance function. Since these examples are labelled with their own ${\hat{bg}}_{t+30}$ values, then a new prediction is formed by averaging (either simply or in a weighted fashion) over the K closest predictions.

#### Support vector regression (radial basis function kernel)

An alternative formulation of SVR is to replace the simple linear kernel with a more complex non-linear kernel. A frequently used choice for this is a radial basis function (RBF) kernel. Since the model itself is non-linear, SVR with an RBF kernel can be surmised to more naturally model the blood glucose dynamics in the data. Consequently, polynomial feature construction is not performed for this approach. The model is also trained differently using the nuSVR algorithm proposed by [20]. It is not expected that models produced via this approach will be interpretable compared to those produced by the linear kernel approach.

#### Multilayer Perceptron (single hidden layer)

**Fig 4:**
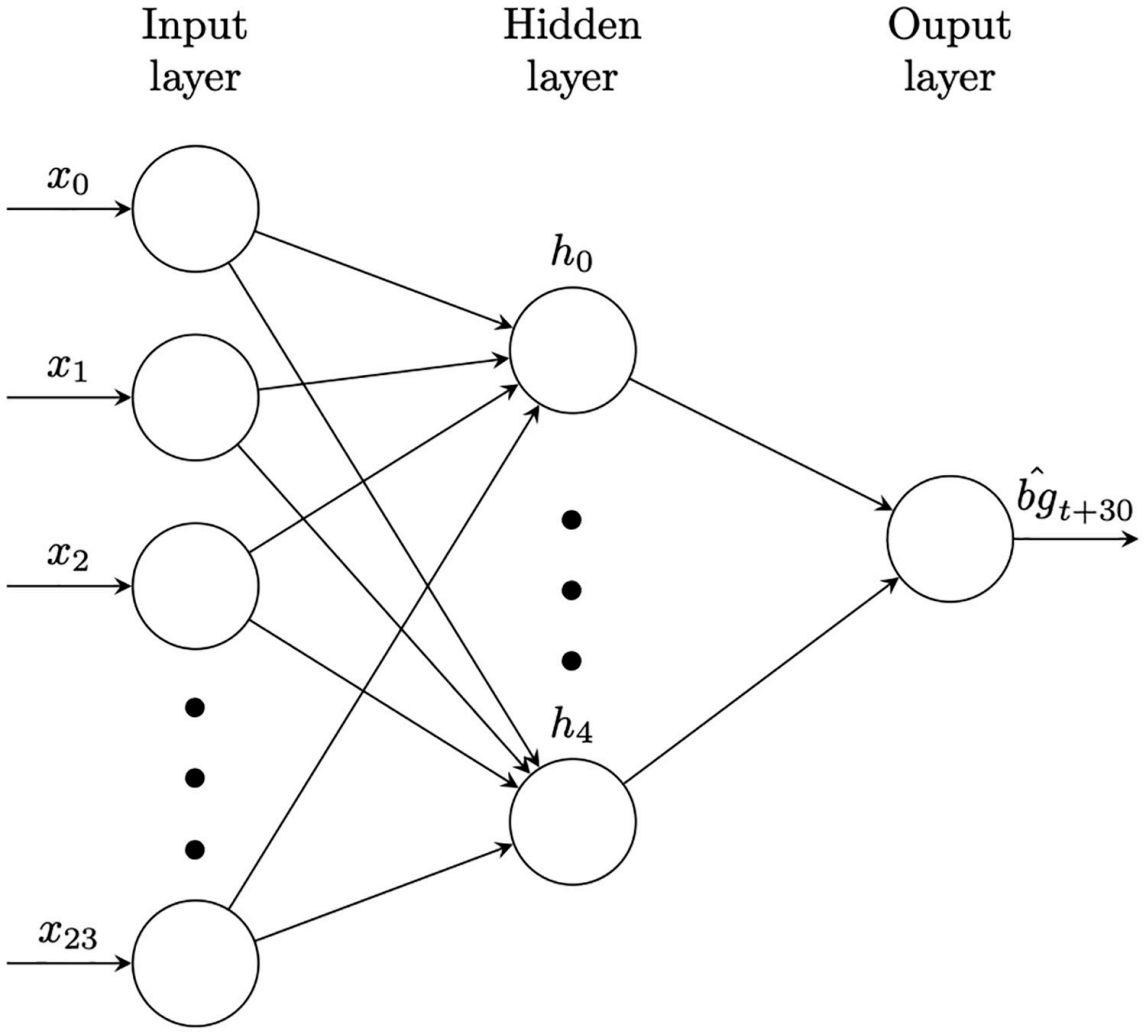
Illustration of a MLP with a single hidden layer of size five.

Multilayer Perceptions (MLPs) are another common method for learning complex non-linear models from data. An MLP for glucose level prediction is depicted by Fig 4. The inputs (CGM sensor readings *x*_0_, *x*_1_, …) are aggregated and non-linearly transformed at intermediate points known as hidden nodes (the *h*_*i*_ nodes in the figure) before being aggregated and transformed again to produce the final prediction ${\hat{bg}}_{t+30}$. In contrast to sparse linear models, MLPs are typically not interpretable due to the complexity of the model.

In an MLP, each edge in the graph represents a distinct numeric parameter that must be optimised by the training process. Note also that some of the edges from the diagram: further edges connecting a constant input to each non-input node are also usually present. The ADAM algorithm [21] is used to train the MLPs.

#### Multilayer Perceptron (two hidden layers)

An MLP capable of learning more complex patterns in the data is one that comprises two hidden layers. A two hidden layer variant is therefore also included in the experiments that follow.

#### Random forest

In contrast to a single decision tree, a random forest [22] is an ensemble of decision trees. In the ensemble, each individual tree makes its own predictions, which are averaged or otherwise aggregated across the entire ensemble to produce a final overall prediction. A key point about the random forest ensemble is that each decision tree is trained on a different randomly selected subset of the input features. This produces a diverse set of individual decision trees. When the predictions of an ensemble are aggregated, the aggregated prediction is generally more accurate than any single decision tree. A distinct drawback is that like MLPs, random forest models are often complex and difficult to interpret due to the number of decision trees and their size. In contrast, a single decision tree such as that shown in Fig 3 is often relatively straightforward to understand provided it is not too large.

#### Gradient boosting

Gradient boosting approaches [23] offer an alternative means of constructing a decision tree ensemble in which each decision tree is trained in sequence instead of independently. The idea is for each consecutive decision tree to learn from the prediction errors of the earlier decisions trees. Predictions from each individual decision tree are then aggregated via weighted summation with appropriately selected weights. Because gradient boosting learns individual decision trees in sequence rather than independently, it can be slower than random forests (in which decision trees can be learned in parallel), but it is often more accurate in practice.

### Oversampling techniques

Oversampling techniques are one method of addressing the data imbalance problem in machine learning. The data imbalance problem occurs because machine learning algorithms make the implicit assumption that the data used for training is balanced across all likely cases. If the data is not balanced, then some algorithms will produce models with significant bias.

To illustrate, suppose there are examples belonging to three sets: hypoglycemic, normoglycemic, and hyperglycemic. An ideal dataset would have a ratio of 33.3% from each range. However, as Fig 2 shows, a more realistic situation is that the groups are imbalanced to a significant degree. For example, 20%:40%:20% is a minor degree of imbalance that most algorithms should cope with. However in the case of Fig 2 the ratio is more extreme at 3.4%:62.5%:34.1%. In this case, the data should be artificially balanced to prevent bias in the model. Two such general approaches are possible: data can be either discarded (which would mean removing the majority of data from the normoglycemia subset and most of the data from the hyperglycemia subset), or synthetic data can be added to the smaller subsets to increase their size. In this paper, this latter group of techniques known collectively as oversampling algorithms is the focus, since initial experiments with discarded data yielded very poor results.

These techniques all apply to labelled data and are usually used for approaching imbalanced classification problems as opposed to regression problems. Therefore additional steps are required to prepare the data before they can be applied in the context of blood glucose level prediction. These steps are discussed in the next section. Note also that in all experimental situations the oversampling is applied only to the training data.

#### Random oversampling

The first technique, random oversampling, creates random duplicates of examples in the dataset. This is performed for the non-majority subsets, which in the case of T1D blood glucose level data is the hypoglycemic and hyperglycemic data. In both cases, examples from these sets are randomly duplicated until the size of both subsets is equal to the size of the normoglycemia subset.

#### Synthetic minority oversampling technique

**Fig 5:**
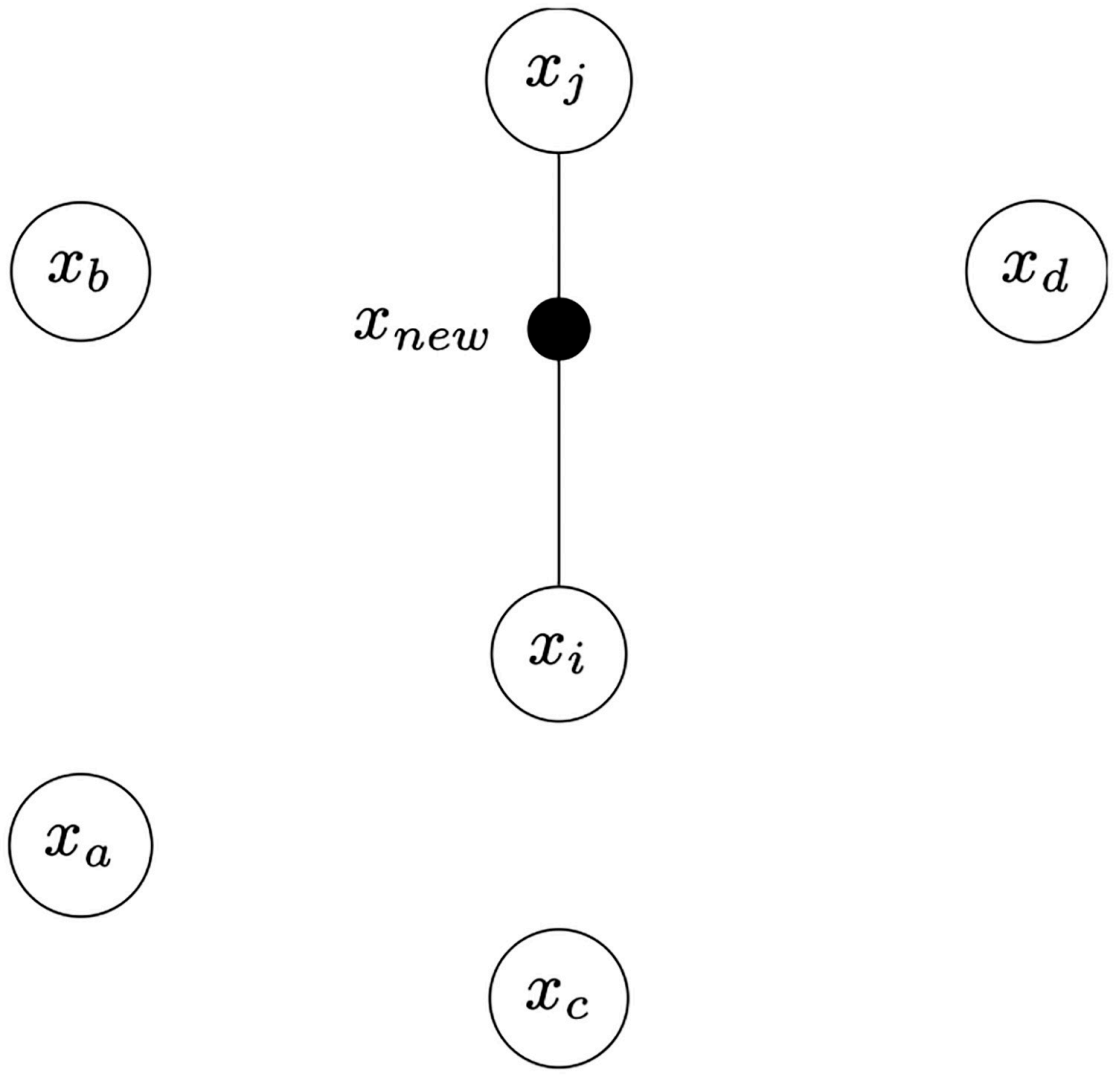
Illustration of SMOTE’s artificial example generation technique.

The second approach, Synthetic Minority Oversampling Technique (SMOTE) [24] is based on the assumption that the minority class examples occur relatively close together in the space of examples. This is usually true when the data contains clusters. Rather than randomly duplicating examples, SMOTE instead creates entirely new “artificial” examples based on this assumption of clustered data. The process involves taking an initial randomly selected minority class example (e.g. an example from the hypoglycemia subset) and then locating nearby similar examples from the same subset. The artificial example is then created by interpolating randomly between the initial example and one of the nearby similar examples, with the amount of interpolation again selected randomly. Fig 5 illustrates this graphically.

In the figure, the nodes represent a group of neighbouring minority class examples in the space of examples that are similar to *x*_*i*_, and *x*_*j*_ is randomly selected from amongst them. The procedure for creating *x*_*new*_ is basic linear interpolation between *x*_*i*_ and *x*_*j*_ as shown by Eq 2, with a randomly selected parameter λ used to control the degree of contribution that each of the examples makes to the new example. In this way new synthetic minority classes can be generated until the classes are more balanced.

$$\begin{array}{c} x_{new}=x_{i}+\lambda \left(x_{j}-x_{i}\right),\lambda \in \left[0,1\right] \end{array}$$ (2)

#### Adaptive synthetic oversampling technique

The third and final approach, Adaptive Synthetic (ADASYN) [25], takes a similar approach as SMOTE. However, ADASYN additionally distinguishes between examples that are “easy” to learn vs. those that are “hard” to learn in the choice of which real examples to use for generating the synthetic examples. The “difficulty” of a real example is determined by comparing the example with its K nearest neighbours (in a fashion similar to the K Nearest Neighbours algorithm described previously), the intuition being that if most of the neighbours are the same, then the example is easy, whereas if most of the neighbours are different, the example if difficult. The fraction of the K nearest neighbours that are from the same minority subset or class are therefore calculated and becomes a proxy for the difficulty of the example.

Next, the synthetic examples are generated from real examples. Whereas SMOTE treats all examples as equal, in ADASYN the number of synthetic instances that a particular minority class example is used to generate is proportional to that example’s difficulty. Therefore, in Fig 5, it can be assumed that the real examples *x*_*i*_ is most likely to be a difficult example.

### Metrics for assessing predictive performance

Two different but complementary metrics are often used in literature relating to CGM data. The first, Mean Absolute Relative Difference (MARD), is a measure of predictive error and is defined by Eq 3.

$$\begin{array}{c} \text{MARD}=\frac{100}{N}\sum_{t=1}^{N}\frac{|{\hat{bg}}_{t+30}-bg_{t+30}|}{bg_{t+30}} \end{array}$$ (3)

In the equation, *N* is the number of predictions made, ${\hat{bg}}_{t+30}$ is the predicted blood glucose level thirty minutes in advance at time *t*, and *bg*_*t*+30_ is the actual blood glucose level that occurred. The errors are converted to a percentage and averaged to give an overall measure of predictive performance. This approach is especially useful for the analysis of blood glucose level predictions because the significance of different absolute prediction errors varies depending on where in the glycemic range the prediction is made. For example, in the hypoglycemic range an error of 10% is much smaller (in absolute mg/dl units) than the same percentage error in the hyperglycemic range. In contrast a significant number of papers in this area use alternative metrics such as root mean squared error (e.g. see the survey paper [11] and recent papers on the blood glucose prediction competition [26]) which are based on absolute units of mg/dl and may result in biased performance estimates.

The second means of assessing predictive performance is the Clarke Error Grid Analysis (EGA) [27], a more clinically oriented approach. Although EGA was originally devised for assessing the accuracy of CGMs themselves by comparing such devices to different reference devices with known accuracy, they can also be used for assessing the predictions made by regression models.

**Fig 6:**
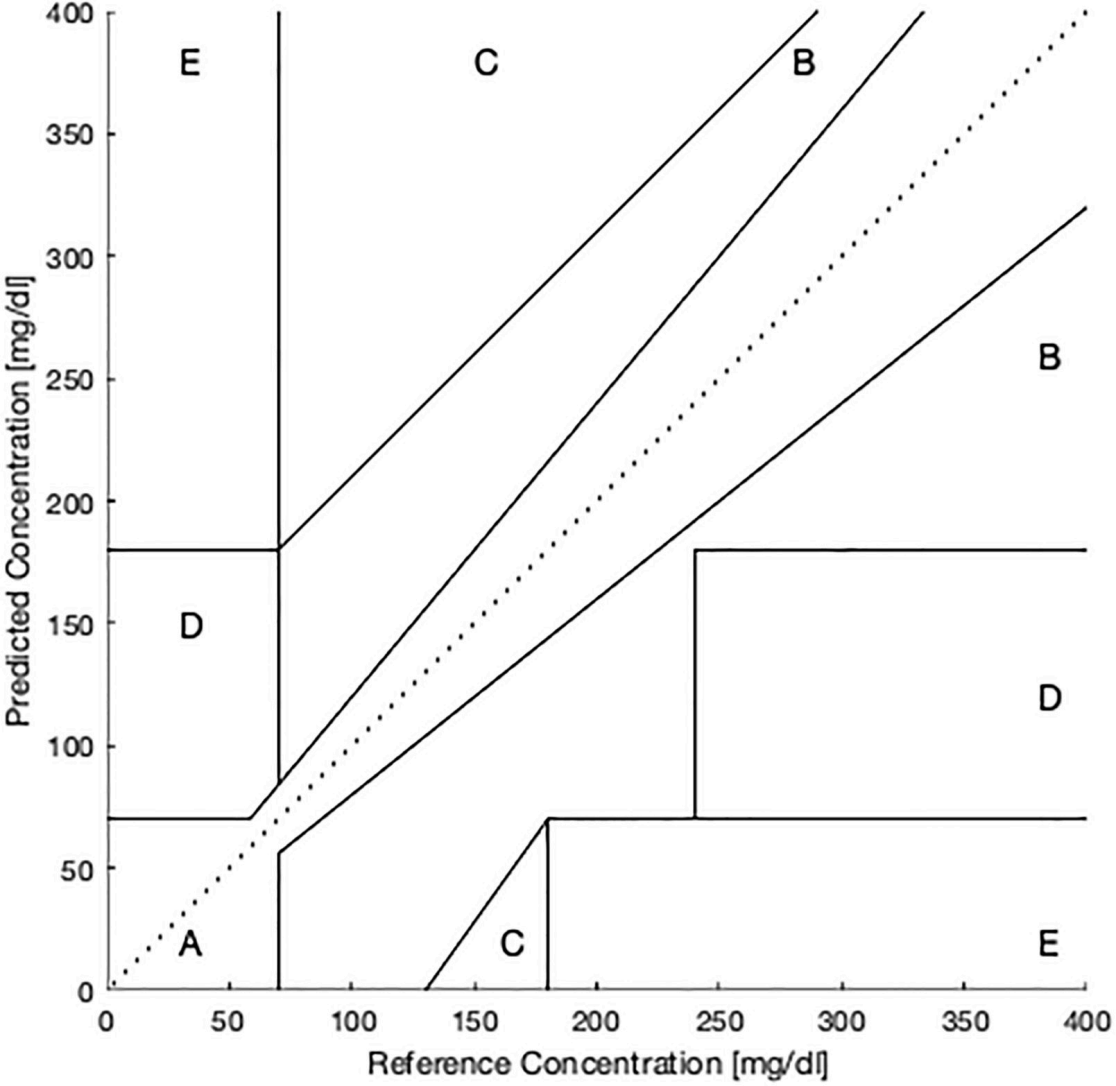
Clarke error grid analysis, reproduced from [28].

Fig 6 depicts the grid used for EGA. As the figure shows, if the prediction/ground truth pairs fall on a 45° line that passes through the origin, then the predictions are perfect. Since most predictions are not perfect, however, the idea behind Clarke EGA is that different errors will have different clinical consequences depending on where they fall in the range as well as their magnitude. In contrast, MARD is only concerned with the size of the error regardless of location.

In a Clarke grid, the most preferred errors lie in Zone A which, as Fig 6 shows, is either the area where the patient will be hypoglycemic (<70 mg/dl blood glucose level) and the prediction is hypoglycemic, or the prediction is within 20% error of the ground truth. Zone B is similar to Zone A, but excludes the hypoglycemic region and permits a larger percentage error. The theory is that the patient would self-administer correct treatment if the error is in Zone A, while the treatment would be “not inappropriate” in Zone B.

Conversely, Zones C, D and E represent prediction errors potentially leading to clinical errors. Specifically, Zone C errors are likely to lead to unnecessary treatments (e.g. the patient will be normoglycemic but predicted to be hyperglycemic) while Zone D and E represent errors that could lead to potentially dangerous treatments (e.g. the patient will be hypoglycemic but is predicted to be hyperglycemic). Therefore a good strategy for model selection involves maximising the number of prediction errors falling into Zone A while minimising the number of errors in the other zones. A disadvantage of this approach is that involves analysing five metrics (i.e. the percentage of errors in each zone) as opposed to one metric, but these numbers provide additional insight.

## Methods

In this section the methods and experimental setup used are described.

### Glycemic-aware metrics and oversampling techniques

Based on the unique nature of the glycemic range and the fact that errors in different parts of the range have considerably different impacts, variants of the standard MARD metric are proposed in this section.

Errors in the hypo-/hyperglycemic ranges are much more significant than errors in the normoglycemic range. The MARD formula from Eq 3 can therefore be generalised by selecting only specific ground truth values *bg*_*t*+30_ and performing the MARD calculation on those. It is assumed that there is some criteria *C* available such that *C*(*bg*_*t*+30_) is true for some ground truth sensor values and false otherwise. A revised version of the MARD formula therefore is given in Eq 4.

$$\begin{array}{c} {\text{MARD}}_{C}=\frac{100}{N_{C}}\sum_{t=1}^{N}\{\begin{array}{c} \frac{|{\hat{bg}}_{t+30}-bg_{t+30}|}{bg_{t+30}}\;\text{if}\;C\left(bg_{t+30}\right)=\text{true}\\ 0\;\text{otherwise} \end{array} \end{array}$$ (4)

In Eq 4, *C* denotes a criteria while *N*_*C*_ is the number of predictions for which the criteria is true, and the rest of the symbols are defined as previously. The criteria is used to split the predictions into three specific subsets: predictions whose ground truth is in the hypoglycemic range (MARD_<70_); predictions whose ground truth is normal (MARD_*norm*_); and predictions whose ground truth is in the hyperglycemic range (MARD_>180_). Overall MARD calculated according to Eq 3 will now be referred to as MARD_*all*_.

Next, glycemic-friendly variants of the oversampling techniques are proposed. Since glucose level prediction is a regression problem, there are no class labels as such. Instead, examples are labelled with a numeric target value *bg*_*t*+30_. Oversampling techniques such as those described previously cannot therefore be applied directly to the data.

To solve this problem, fake or “pseudolabels” for the examples are generated and used. The pseudolabels, like the MARD variants above, can be specifically designed for the glycemic range by discretising the target value into three parts (i.e. hypo-, normo- and hyperglycemic). The range then determines the pseudolabel. Table 2 illustrates the process for six examples from the dataset. The pseudolabels can be discarded after the oversampling procedure is complete.

**Table 2:** Examples of feature vectors constructed from the CGM traces. Features *x*_1_ to *x*_24_ are consecutive CGM sensor readings occurring over a period of 120 minutes; *bg*_*t*+30_ is the glucose value observed 30 minutes after *x*_24_. The pseudolabel for each example, which is only used if an oversampling method is employed, is also shown.

*x*_1_	*x*_2_	…	*x*_23_	*x*_24_	*bg*_*t*+30_	Pseudolabel
215	209	…	158	151	125	L_normal_
149	137	…	109	113	133	L_normal_
165	163	…	171	174	187	L_>180_
154	158	…	217	217	234	L_>180_
65	70	…	71	70	60	L_<70_
105	109	…	64	64	65	L_<70_

Note that a key novel difference between the oversampling proposed here and standard oversampling techniques is that the numeric targets (i.e. the *bg*_*t*+30_ values) are also synthetically generated with the features. In contrast, standard approaches usually only synthetically generate the features of each example while keeping the labels fixed.

### Experimental setup

To evaluate the range of machine learning algorithms described in the previous section against the Ohio T1DM dataset, the following experiment was devised. Firstly the ten machine learning algorithms (dummy, Lasso, decision tree, etc) were combined with four oversampling options. The four oversampling options were no oversampling (as a baseline), random oversampling, SMOTE, and ADASYN. This yielded a total of forty different combinations.

Next, each of the forty combinations was trained six times, against each individual patient’s training data. The resulting models were then tested against each patient’s corresponding test dataset (see Table 1 as a reminder of the size of each dataset). Thus, a total of 240 train/test experiments were performed.

Once test data predictions were recorded, performance metrics were calculated. The metrics were MARD_*all*_, MARD_<70_,MARD_>180_ and MARD_*norm*_, along with the percentage frequencies of prediction errors falling into all five zones of the Clarke error grid.

In terms of preprocessing and hyperparameters used, feature-wise standardisation (with the mean and standard deviation calculated based on the training data) was used to scale the data before any of the machine learning algorithms were applied. For the Lasso model, five-fold cross validation on the training data was used to select the optimal alpha parameter; for the Lasso, SVR, and MLP models, the number of iterations was set at 100,000; and for the decision tree ensembles, the number of trees was set at 500. The MLP models additionally had the size of each hidden layer fixed at five units, and the activation function for each hidden node was tanh. All other parameters and settings were defaults as set in the sci-kit learn [29] v 0.20.3 and imbalanced learn [30] v 0.4.3 APIs.

## Results

Complete and detailed tables of results with mean and standard deviation performances by algorithm, oversampler and metric are given in S1 Appendix. In this section, summarised results are primarily presented along with the results of various statistical tests.

However, it is prudent to note that an examination of the raw result tables shows that some zones of the Clarke error grid are not useful for the purposes of selecting the best model. To illustrate, consider Table D in S1 Appendix. In this table, the dummy method records 0% frequency of prediction errors in EGA Zones C and E, which is the lower than all of the nine other options in the table. Under the assumption that a lower error rate in these zones is “safer”, this would imply that the dummy classifier has reasonably good predictive performance. However, the error rates in Zone A show that the dummy classifier achieves only 33% compared to 74% for the next best method. In other words, selecting a model based on the frequencies of errors in Zones C and E is unreliable and these metrics should be given less weight than the other metrics.

Table 3 gives the first summary of the results. In this table, all of the predictive model/oversampling combinations are ranked by overall MARD_*all*_ and averaged across patient. The table shows the top five algorithms in the ranking. Surprisingly, all of the best models are linear models, but trained using the additional polynomial features. Linear SVR and no oversampling achieves the best overall average value for MARD_*all*_.

**Table 3:** Top five model and resampler combinations based on overall MARD.

Combination	MARD_*all*_
Lin. SVR	10.19
Lasso + SMOTE	10.29
Lasso + Random	10.31
Lin. SVR + SMOTE	10.37
Lin. SVR + Random	10.39

The story is different when considering the proposed alternative range-specific MARD metrics, however. Table 4 presents the same ranking, but this time for the MARD_<70_, MARD_>180_ and MARD_*norm*_ metrics. The table shows that linear SVR’s excellent overall performance is primarily due to accurate predictions being made in the normal range. In contrast, outside the normal range (in both the hypo- and hyperglycemic subranges) an MLP with two hidden layers combined with some form of oversampling records the best prediction accuracy.

**Table 4:** Top five model and resampler combinations based on alternative MARD metrics.

Combination	MARD_>180_	Combination	MARD_*norm*_
MLP(5,5) + Random	8.19	Lin. SVR	10.88
MLP(5,5) + SMOTE	8.23	MLP(5,5)	10.94
MLP(5) + SMOTE	8.27	GB	11.12
MLP(5) + Random	8.31	MLP(5)	11.17
MLP(5,5)	8.45	Lasso + Random	11.26
Combination	MARD_<70_		
MLP(5,5) + ADASYN	12.46		
MLP(5,5) + SMOTE	13.20		
MLP(5) + ADASYN	14.02		
MLP(5,5) + Random	14.18		
MLP(5) + SMOTE	14.85		

To further investigate this, a standard paired t-test comparing the six MARD_<70_ values of the best MLP classifier (MLP(5,5)+ADASYN) against the linear SVR approach was performed. The linear SVR approach performed well overall (see Table 3), but poorly according the MARD_<70_ metric, having an average error of 26.01 while the table shows that the best MLP approach scored 12.46. This difference in mean performance was found significant at 95% confidence with *p* = 0.025.

A similar comparison using the MARD_>180_ metric was also performed. Again, linear SVR was compared to the best MLP classifier (MLP(5,5)+ADASYN this time) where the mean errors were 8.74 and 8.19 respectively (see S1 Appendix for linear SVR’s MARD_>180_ result). This time no significant difference at 95% confidence was found between the means with *p* = 0.1795. The difference is significant at an 80% level of confidence, however.

Next, results from the Clarke EGA were examined. The top ranked algorithms as determined by percentage frequencies are given by Table 5. For Zone A, a higher percentage is better since this represents the most benign class of error; for other zones, a lower rate is better. This table tells a similar story to that of the MARD-based analysis: linear SVR achieves the best results in terms of Zone A and B errors, while the MLP approach with oversampling is most effective at minimising the more dangerous Zone D errors. For the Zone C and E errors, results from the dummy method were excluded and consequently decision trees and KNN achieve the best performance in these zones. However, like the dummy method, decision trees and KNN perform poorly according to the other metrics, and therefore the Zone C/E frequencies are excluded from further analysis.

**Table 5:** Top five model and resampler combinations based on EGA metrics (excludes the dummy predictor).

Combination	EGA_*A*_	Combination	EGA_*B*_
Lin. SVR	87.17	Lin. SVR	11.59
Lasso + Random	86.97	MLP(5,5)	11.76
Lasso + SMOTE	86.94	Lasso	11.86
Lasso + ADASYN	86.89	MLP(5)	12.01
Lin. SVR + SMOTE	86.73	GB	12.15
Combination	EGA_*C*_	Combination	EGA_*D*_
KNN + Random	0.00	MLP(5) + Random	0.51
KNN + ADASYN	0.00	MLP(5) + ADASYN	0.52
KNN	0.01	MLP(5) + SMOTE	0.55
KNN + SMOTE	0.01	NuSVR + Random	0.57
NuSVR + ADASYN	0.02	NuSVR + SMOTE	0.57
Combination	EGA_*E*_		
DT	0.00		
KNN	0.00		
KNN + Random	0.00		
KNN + SMOTE	0.00		
KNN + ADASYN	0.00		

A Friedman test [31] comparing all forty machine learning/oversampler combinations across all six patients was also performed. The Friedman test is a statistical test for determining if all the prediction methods perform the same or differently. The test was performed once for each metric, and the *p*-values are given in Table 6. These values indicate that in all cases except MARD_<70_, there is a strongly significant difference (more than 99% confidence) between the predictive accuracies. For the MARD_<70_ case, confidence is more than 80% but less than 95%. A further posthoc Nemenyi test [32] was also performed in order to isolate the best subset of equivalent methods, but this test failed to produce significant results. This is most likely due to the small number of patients in the dataset.

**Table 6:** Friedman test *p*-values after testing for the null hypothesis that all classifiers perform equally well.

Metric	*p* value
MARD_*all*_	1.573e-05
MARD_<70_	0.1309
MARD_*norm*_	0.0001133
MARD_>180_	0.001298
EGA_*A*_	0.004514
EGA_*B*_	0.0004646
EGA_*D*_	0.007071

**Fig 7:**
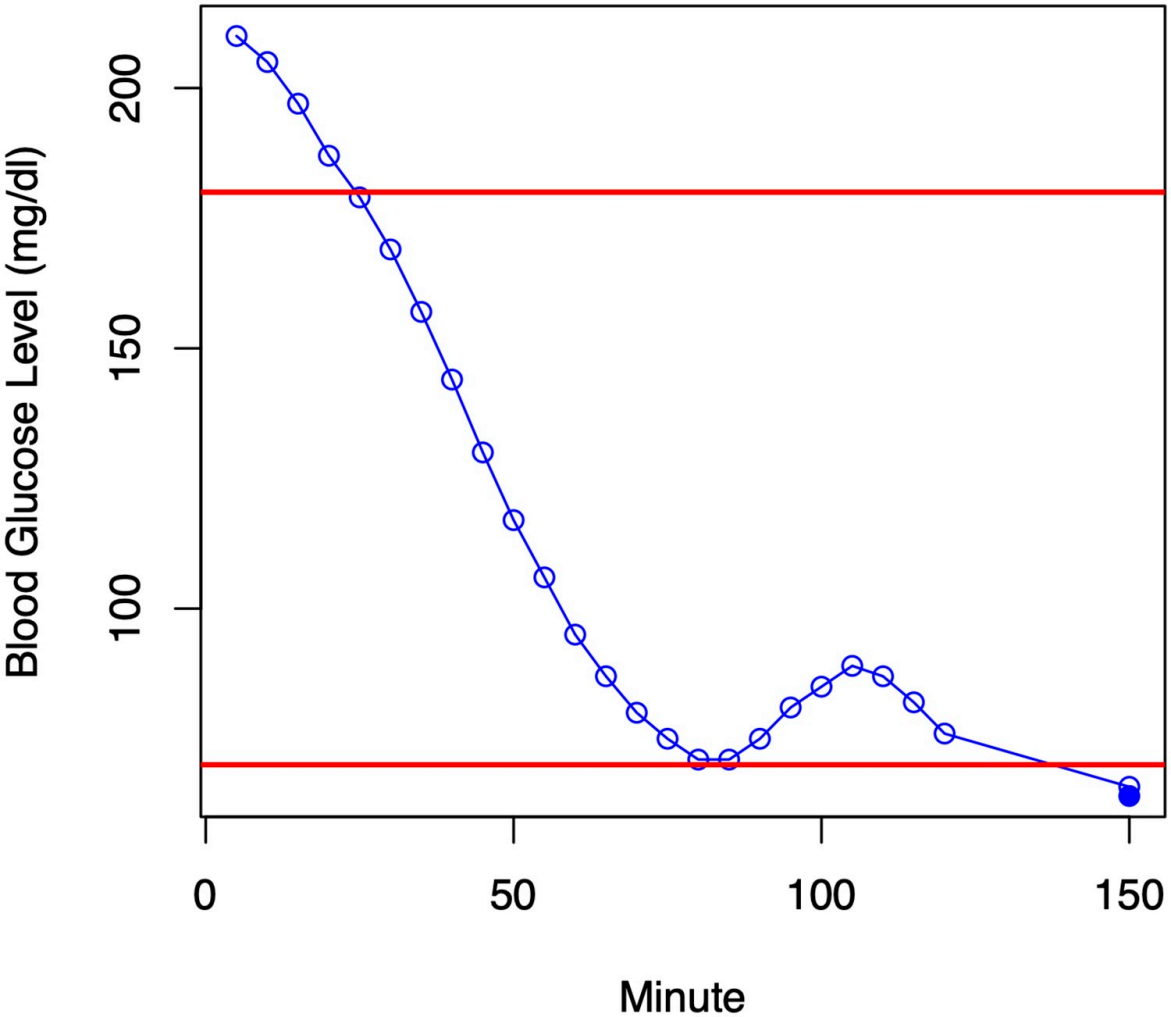
Examples of a prediction made by linear SVR for one patient. The first 120 minutes of the plot (unfilled circles) are the inputs to the model; the last reading at 150 minutes is the prediction (unfilled) and what actually happened (filled). This figure depicts a Type A error.

**Fig 8:**
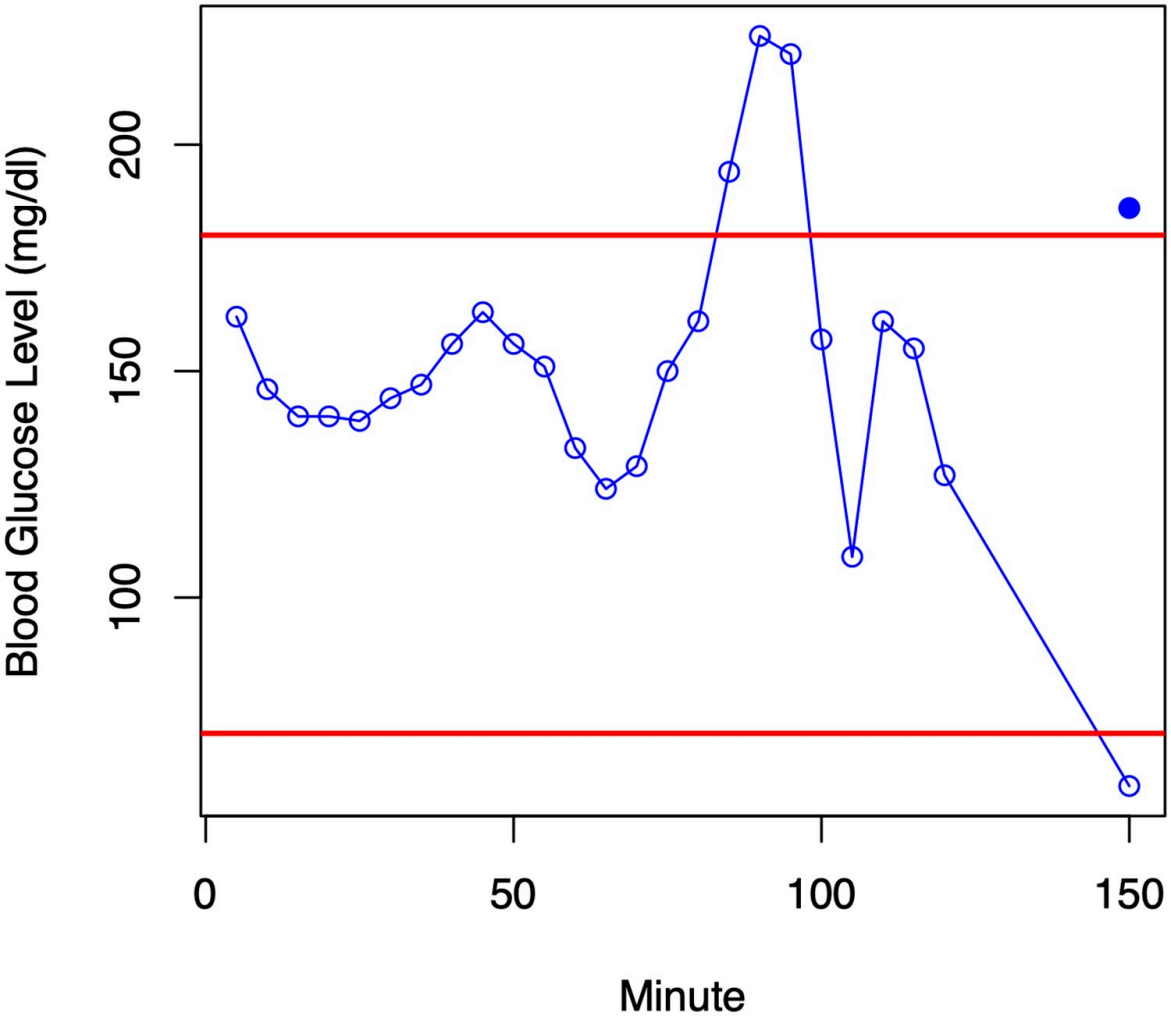
Similar example to that depicted in Fig 7, but depicting a Type E error.

Finally, some examples of predictions made by the linear SVR method are shown in Figs 7 and 8. In both situations, the prediction is that the patient will be hypoglycemic in the next thirty minutes; in one case the prediction is correct (a Type A error) and in the second case it is seriously wrong (a Type E error). One possible explanation for the Type E error is that the patient ate something in the interval between the end of the prediction window and the prediction point thirty minutes later.

## Discussion

There are two main takeaways from the results presented here. Firstly, relying on a single overall metric for model selection (often a standard approach in machine learning) is unlikely to be sufficient since it will not reveal information on the predictive accuracy of a method in subranges that are rarely visited. The main illustration here is the <70 mg/dl range where performance of the best overall method was significantly poorer than another method. Analysis should instead follow an approach similar to that performed here: break the overall error metric down into its constituent parts and analyse each constituent part separately. Alternative clinical metrics such as those derived from Clarke EGA are also extremely useful and could even be the primary method of evaluation.

The second important takeaway is that the datasets used for training the model will be imbalanced, and it is shown here that techniques addressing imbalance improve accuracy. Again, prior works will often ignore this issue and therefore the results they present are most likely biased.

To conclude, some recommendations for the most appropriate approaches for predicting blood glucose levels can be made. The results show that if the application is hypoglycemia alerting, an MLP trained in conjunction with an oversampling method produces the best accuracy. On the other hand, for general forecasting of blood glucose levels over the ≥ 70 mg/dl range, a linear SVR trained on prediction window sensor readings plus polynomial features calculated from the original readings is the closest approach to optimal. Thus, the choice of best algorithm to use depends on where in the glycemic range it will be applied. In contrast, most other works in the literature do not perform this kind of breakdown analysis of predictions across different regions of the glycemic range. A strong recommendation therefore for future work is to analyse model errors by subrange, using both numeric metrics such as MARD and clinical metrics such as EGA. Additionally, if general purpose prediction across the entire range is required, researchers could consider the use of multiple models, each model optimised for its own particular glycemic subrange.

## Supporting information

S1 Appendix Complete and detailed tables of results with mean and standard deviation performances by algorithm, oversampler and metric. (PDF)

## References

[1] DiMeglio LA, Evans-Molina C, Oram RA. Type 1 diabetes. The Lancet. 2018;391(10138):2449–2462. 10.1016/S0140-6736(18)31320-5 PMC6661119 29916386

[2] Lind M, Polonsky W, Hirsch IB, Heise T, Bolinder J, Dahlqvist S, et al Continuous Glucose Monitoring vs Conventional Therapy for Glycemic Control in Adults With Type 1 Diabetes Treated With Multiple Daily Insulin Injections: The GOLD Randomized Clinical Trial. JAMA. 2017;317(4):379–387. 10.1001/jama.2016.19976 28118454

[3] Garg SK, Weinzimer SA, Tamborlane WV, Buckingham BA, Bode BW, Bailey TS, et al Glucose Outcomes with the In-Home Use of a Hybrid Closed-Loop Insulin Delivery System in Adolescents and Adults with Type 1 Diabetes. Diabetes Technology & Therapeutics. 2017;19(3):155–163. 10.1089/dia.2016.0421 28134564 PMC5359676

[4] Miller KM, Foster NC, Beck RW, Bergenstal RM, DuBose SN, DiMeglio LA, et al Current State of Type 1 Diabetes Treatment in the U.S.: Updated Data From the T1D Exchange Clinic Registry. Diabetes Care. 2015;38(6):971–978. 10.2337/dc15-0078 25998289

[5] Rama Chandran S, Tay WL, Lye WK, Lim LL, Ratnasingam J, Tan ATB, et al Beyond HbA1c: Comparing Glycemic Variability and Glycemic Indices in Predicting Hypoglycemia in Type 1 and Type 2 Diabetes. Diabetes Technology & Therapeutics. 2018;20(5):353–362. 10.1089/dia.2017.0388 29688755

[6] Abraham MB, Nicholas JA, Smith GJ, Fairchild JM, King BR, Ambler GR, et al Reduction in Hypoglycemia With the Predictive Low-Glucose Management System: A Long-Term Randomized Controlled Trial in Adolescents With Type 1 Diabetes. Diabetes Care. 2017 10.2337/dc17-1604 29191844

[7] Del Favero S, Place J, Kropff J, Messori M, Keith-Hynes P, Visentin R, et al Multicenter outpatient dinner/overnight reduction of hypoglycemia and increased time of glucose in target with a wearable artificial pancreas using modular model predictive control in adults with type 1 diabetes. Diabetes, Obesity and Metabolism. 2015;17(5):468–476. 10.1111/dom.12440 25600304

[8] Bruen D, Delaney C, Florea L, Diamond D. Glucose sensing for diabetes monitoring: recent developments. Sensors. 2017;17(8):1866 10.3390/s17081866 PMC5579887 28805693

[9] Kovatchev B. Automated closed-loop control of diabetes: the artificial pancreas. Bioelectronic Medicine. 2018;4(1):14 10.1186/s42234-018-0015-6 PMC7098217 32232090

[10] Oviedo S, Vehí J, Calm R, Armengol J. A review of personalized blood glucose prediction strategies for T1DM patients. International Journal for Numerical Methods in Biomedical Engineering. 2017;33(6):e2833 10.1002/cnm.2833 27644067

[11] Woldaregay AZ, Årsand E, Walderhaug S, Albers D, Mamykina L, Botsis T, et al Data-driven modeling and prediction of blood glucose dynamics: Machine learning applications in type 1 diabetes. Artificial Intelligence in Medicine. 2019;98:109–134. 10.1016/j.artmed.2019.07.007. 31383477

[12] Marling C, Bunescu R. Benchmark Machine Learning Approaches with Classical Time Series Approaches on the Blood Glucose Level Prediction Challenge. In: Proc. of the 3rd International Workshop on Knowledge Discovery in Healthcare Data; 2018. p. 97–102.

[13] Branco P, Torgo L, Ribeiro RP. A Survey of Predictive Modeling on Imbalanced Domains. ACM Comput Surv. 2016;49(2):31:1–31:50. 10.1145/2907070

[14] Marling C, Bunescu R. The OhioT1DM Dataset For Blood Glucose Level Prediction. In: Proc. of the 3rd International Workshop on Knowledge Discovery in Healthcare Data; 2018. p. 60–63.

[15] Danne T, Nimri R, Battelino T, Bergenstal RM, Close KL, DeVries JH, et al International Consensus on Use of Continuous Glucose Monitoring. Diabetes Care. 2017;40(12):1631–1640. 10.2337/dc17-1600 29162583 PMC6467165

[16] Friedman J, Hastie T, Tibshirani R. Regularization Paths for Generalized Linear Models via Coordinate Descent. Journal of Statistical Software, Articles. 2010;33(1):1–22. PMC2929880 20808728

[17] Kim SJ, Koh K, Lustig M, Boyd S, Gorinevsky D. An Interior-Point Method for Large-Scale L_1_-Regularized Least Squares. IEEE Journal of Selected Topics in Signal Processing. 2007;1(4):606–617. 10.1109/JSTSP.2007.910971

[18] Smola AJ, Schölkopf B. A tutorial on support vector regression; 2004.

[19] Breiman L, Friedman JH, Olshen RA, Stone CJ. Classification and Regression Trees. Monterey, CA: Wadsworth and Brooks; 1984.

[20] Schölkopf B, Smola AJ, Williamson RC, Bartlett PL. New Support Vector Algorithms. Neural Comput. 2000;12(5):1207–1245. 10.1162/089976600300015565 10905814

[21] Kingma DP, Ba J. ADAM: A method for stochastic optimization. arXiv preprint arXiv:14126980. 2014.

[22] Breiman L. Random forests. Machine Learning. 2001;45(1):5–32. 10.1023/A:1010933404324

[23] Friedman JH. Stochastic Gradient Boosting. Comput Stat Data Anal. 2002;38(4):367–378. 10.1016/S0167-9473(01)00065-2

[24] Chawla NV, Bowyer KW, Hall LO, Kegelmeyer WP. SMOTE: Synthetic Minority Over-sampling Technique. J Artif Int Res. 2002;16(1):321–357.

[25] He H, Bai Y, Garcia EA, Li S. ADASYN: Adaptive synthetic sampling approach for imbalanced learning. In: 2008 IEEE International Joint Conference on Neural Networks (IEEE World Congress on Computational Intelligence); 2008. p. 1322–1328.

[26] Zhu T, Li K, Herrero P, Chen J, Georgiou P. A Deep Learning Algorithm for Personalized Blood Glucose Prediction. In: Proc. of the 3rd International Workshop on Knowledge Discovery in Healthcare Data; 2018. p. 64–78.

[27] Clarke WL, Cox D, Gonder-Frederick LA, Carter W, Pohl SL. Evaluating clinical accuracy of systems for self-monitoring of blood glucose. Diabetes care. 1987;10(5):622–628. 10.2337/diacare.10.5.622 3677983

[28] Clarke error grid analysis https://commons.wikimedia.org/wiki/File:Clarkeerrorgrid.gif.

[29] Pedregosa F, Varoquaux G, Gramfort A, Michel V, Thirion B, Grisel O, et al Scikit-learn: Machine Learning in Python. Journal of Machine Learning Research. 2011;12:2825–2830.

[30] Lemaître G, Nogueira F, Aridas CK. Imbalanced-learn: A Python Toolbox to Tackle the Curse of Imbalanced Datasets in Machine Learning. Journal of Machine Learning Research. 2017;18(17):1–5.

[31] Hollander M, Wolfe D, Chicken E. Nonparametric Statistical Methods, 3rd Edition; 2015.

[32] Sachs L. Angewandte Statistik, 8th Ed; 1997.

