# Supplementary material for: Glycemic-aware metrics and oversampling techniques for predicting blood glucose levels using machine learning
Source: PLoS One. 2019 Dec 2;14(12):e0225613. doi: 10.1371/journal.pone.0225613 (PMC6886807; doi:10.1371/journal.pone.0225613)
Supplement: S1 Appendix — (PDF) [file pone.0225613.s001.pdf]

Model	$MARD_{all}$	$MARD_{>180}$	$MARD_{norm}$	$MARD_{<70}$
Dummy	32.29 ± 7.02	27.62 ± 5.37	32.08 ± 10.71	160.27 ± 28.68
Lasso	10.60 ± 2.65	8.52 ± 1.92	11.36 ± 1.81	34.70 ± 8.82
Lin. SVR	10.19 ± 2.39	8.74 ± 2.05	10.88 ± 1.74	26.01 ± 7.17
DT	15.59 ± 3.69	12.38 ± 2.53	17.09 ± 2.81	49.48 ± 26.71
KNN	14.46 ± 3.23	11.53 ± 2.19	15.43 ± 2.04	44.60 ± 9.53
NuSVR	12.59 ± 3.02	10.29 ± 2.12	12.64 ± 1.70	54.16 ± 7.90
MLP(5)	10.67 ± 2.61	8.53 ± 1.85	11.17 ± 1.60	42.29 ± 7.67
MLP(5,5)	10.49 ± 2.50	8.45 ± 1.80	10.94 ± 1.69	38.78 ± 8.96
RF	11.14 ± 2.61	9.11 ± 1.92	11.64 ± 1.69	39.68 ± 8.21
GB	10.87 ± 2.65	8.95 ± 1.85	11.12 ± 1.64	40.47 ± 7.77

Table A: MARD±sd test results averaged over patient for regressors with no oversampling.

Model	EGA_A	EGA_B	EGA_C	EGA_D	EGA_E
Dummy	41.33 ± 8.74	49.07 ± 10.60	0.06 ± 0.15	9.47 ± 5.96	0.07 ± 0.18
Lasso	86.22 ± 6.16	11.86 ± 4.63	0.06 ± 0.09	1.84 ± 1.74	0.01 ± 0.03
Lin. SVR	87.17 ± 5.51	11.59 ± 4.37	0.08 ± 0.13	1.12 ± 1.14	0.03 ± 0.06
DT	74.09 ± 7.83	23.66 ± 6.44	0.36 ± 0.33	1.82 ± 1.19	0.08 ± 0.11
KNN	76.43 ± 7.42	21.23 ± 5.68	0.12 ± 0.15	2.22 ± 1.85	0.00 ± 0.00
NuSVR	80.89 ± 6.93	16.35 ± 4.72	0.01 ± 0.02	2.76 ± 2.45	0.00 ± 0.00
MLP(5)	85.80 ± 6.18	12.01 ± 4.29	0.04 ± 0.04	2.15 ± 2.04	0.01 ± 0.02
MLP(5,5)	86.12 ± 5.69	11.76 ± 3.99	0.03 ± 0.06	2.09 ± 1.89	0.00 ± 0.00
RF	84.99 ± 6.36	13.10 ± 4.86	0.03 ± 0.06	1.88 ± 1.64	0.00 ± 0.00
GB	85.66 ± 6.20	12.15 ± 4.31	0.06 ± 0.09	2.13 ± 1.96	0.00 ± 0.00

Table B: EGA±sd test results averaged over patient for regressors with no oversampling.

Model	$MARD_{all}$	$MARD_{>180}$	$MARD_{norm}$	$MARD_{<70}$
Dummy	31.89 ± 5.31	37.05 ± 2.71	23.01 ± 4.47	126.26 ± 19.87
Lasso	10.31 ± 2.27	8.50 ± 1.55	11.26 ± 1.77	18.78 ± 6.80
Lin. SVR	10.39 ± 2.37	8.74 ± 1.82	11.29 ± 1.98	18.16 ± 7.09
DT	15.65 ± 3.60	12.65 ± 2.53	16.80 ± 2.69	40.06 ± 7.53
KNN	15.15 ± 3.51	11.96 ± 2.46	16.70 ± 2.53	38.43 ± 7.23
NuSVR	11.93 ± 2.26	9.74 ± 1.61	13.27 ± 2.00	17.50 ± 6.49
MLP(5)	10.56 ± 2.18	8.31 ± 1.51	11.95 ± 1.70	14.96 ± 4.12
MLP(5,5)	10.96 ± 2.41	8.19 ± 1.43	12.74 ± 2.25	14.18 ± 4.23
RF	11.28 ± 2.63	9.16 ± 1.98	11.94 ± 1.69	37.16 ± 5.88
GB	10.96 ± 2.34	8.49 ± 1.59	12.22 ± 1.59	21.65 ± 6.79

Table C: MARD±sd test results averaged over patient for regressors with random oversampling.

Model	EGA_A	EGA_B	EGA_C	EGA_D	EGA_E
Dummy	32.71 ± 6.95	51.17 ± 6.16	0.00 ± 0.00	16.12 ± 12.24	0.00 ± 0.00
Lasso	86.97 ± 5.04	12.18 ± 4.48	0.08 ± 0.09	0.75 ± 0.59	0.03 ± 0.03
Lin. SVR	86.70 ± 5.13	12.47 ± 4.68	0.12 ± 0.19	0.67 ± 0.52	0.03 ± 0.06
DT	74.14 ± 7.39	23.72 ± 6.20	0.31 ± 0.24	1.80 ± 1.26	0.04 ± 0.05
KNN	74.51 ± 8.09	23.60 ± 6.68	0.17 ± 0.21	1.71 ± 1.40	0.01 ± 0.02
NuSVR	81.98 ± 5.46	17.30 ± 4.99	0.00 ± 0.00	0.73 ± 0.59	0.00 ± 0.00
MLP(5)	85.97 ± 5.18	13.38 ± 4.71	0.04 ± 0.07	0.57 ± 0.47	0.04 ± 0.05
MLP(5,5)	84.52 ± 6.03	14.87 ± 5.65	0.05 ± 0.07	0.51 ± 0.44	0.06 ± 0.08
RF	84.50 ± 6.25	13.74 ± 4.85	0.05 ± 0.09	1.71 ± 1.54	0.00 ± 0.00
GB	85.20 ± 5.58	13.73 ± 4.71	0.05 ± 0.07	1.01 ± 0.93	0.01 ± 0.02

Table D: EGA±sd test results averaged over patient for regressors with random oversampling.

Model	$MARD_{all}$	$MARD_{>180}$	$MARD_{norm}$	$MARD_{<70}$
Dummy	31.90 ± 5.31	37.04 ± 2.73	23.01 ± 4.47	126.28 ± 19.84
Lasso	10.29 ± 2.26	8.46 ± 1.54	11.26 ± 1.76	18.77 ± 7.33
Lin. SVR	10.37 ± 2.38	8.69 ± 1.79	11.29 ± 1.97	18.01 ± 7.98
DT	15.59 ± 3.50	12.47 ± 2.53	17.22 ± 2.67	33.47 ± 4.88
KNN	15.01 ± 3.40	11.74 ± 2.33	16.77 ± 2.57	34.55 ± 7.80
NuSVR	11.87 ± 2.26	9.71 ± 1.63	13.18 ± 1.97	17.81 ± 6.65
MLP(5)	10.56 ± 2.22	8.27 ± 1.52	12.01 ± 1.76	14.85 ± 5.49
MLP(5,5)	10.84 ± 2.26	8.23 ± 1.47	12.64 ± 1.87	13.20 ± 5.26
RF	11.16 ± 2.54	9.06 ± 1.97	12.06 ± 1.74	33.31 ± 10.56
GB	10.90 ± 2.31	8.59 ± 1.65	12.10 ± 1.61	22.70 ± 3.98

Table E: MARD±sd test results averaged over patient for regressors with SMOTE oversampling.

Model	EGA_A	EGA_B	EGA_C	EGA_D	EGA_E
Dummy	32.86 ± 7.08	51.02 ± 6.19	0.00 ± 0.00	16.12 ± 12.24	0.00 ± 0.00
Lasso	86.94 ± 5.05	12.22 ± 4.44	0.10 ± 0.11	0.72 ± 0.60	0.03 ± 0.03
Lin. SVR	86.73 ± 5.26	12.47 ± 4.77	0.12 ± 0.16	0.65 ± 0.51	0.02 ± 0.03
DT	73.32 ± 8.15	24.76 ± 7.07	0.26 ± 0.18	1.62 ± 1.09	0.04 ± 0.06
KNN	74.80 ± 8.15	23.49 ± 6.94	0.18 ± 0.20	1.50 ± 1.19	0.03 ± 0.04
NuSVR	82.16 ± 5.52	17.11 ± 5.07	0.01 ± 0.02	0.72 ± 0.56	0.00 ± 0.00
MLP(5)	85.99 ± 5.34	13.34 ± 4.84	0.03 ± 0.05	0.57 ± 0.51	0.08 ± 0.09
MLP(5,5)	85.30 ± 5.30	14.01 ± 4.90	0.04 ± 0.07	0.55 ± 0.39	0.10 ± 0.11
RF	84.65 ± 6.22	13.94 ± 5.02	0.05 ± 0.07	1.36 ± 1.26	0.00 ± 0.00
GB	85.44 ± 5.35	13.60 ± 4.60	0.05 ± 0.08	0.90 ± 0.79	0.00 ± 0.00

Table F: EGA±sd test results averaged over patient for regressors with SMOTE oversampling.

Model	$MARD_{all}$	$MARD_{>180}$	$MARD_{norm}$	$MARD_{<70}$
Dummy	31.82 ± 4.45	38.73 ± 2.09	22.40 ± 3.75	120.31 ± 19.83
Lasso	10.52 ± 2.34	8.66 ± 1.53	11.42 ± 1.91	20.22 ± 7.69
Lin. SVR	10.57 ± 2.42	8.85 ± 1.89	11.54 ± 1.96	17.87 ± 6.91
DT	15.86 ± 3.66	12.72 ± 2.54	17.46 ± 3.06	37.59 ± 8.44
KNN	14.90 ± 3.44	11.29 ± 1.96	16.76 ± 2.74	34.29 ± 9.12
NuSVR	11.79 ± 1.91	10.00 ± 1.59	12.78 ± 1.56	18.09 ± 9.67
MLP(5)	11.04 ± 2.61	8.47 ± 1.68	12.64 ± 2.21	14.02 ± 5.03
MLP(5,5)	11.30 ± 2.66	8.46 ± 1.35	13.03 ± 2.55	12.46 ± 6.12
RF	11.27 ± 2.56	8.94 ± 1.84	12.26 ± 1.73	30.26 ± 6.10
GB	11.37 ± 2.64	8.62 ± 1.74	12.69 ± 1.91	22.83 ± 9.37

Table G: MARD±sd test results averaged over patient for regressors with ADASYN oversampling.

Model	EGA_A	EGA_B	EGA_C	EGA_D	EGA_E
Dummy	32.22 ± 6.16	51.65 ± 6.41	0.00 ± 0.00	16.12 ± 12.24	0.00 ± 0.00
Lasso	86.89 ± 5.02	12.18 ± 4.30	0.09 ± 0.09	0.82 ± 0.73	0.03 ± 0.03
Lin. SVR	86.49 ± 5.00	12.56 ± 4.33	0.27 ± 0.31	0.60 ± 0.48	0.08 ± 0.12
DT	73.46 ± 8.11	24.43 ± 6.78	0.39 ± 0.37	1.64 ± 1.23	0.08 ± 0.08
KNN	75.42 ± 7.71	22.86 ± 6.46	0.15 ± 0.18	1.52 ± 1.24	0.06 ± 0.06
NuSVR	82.20 ± 4.63	16.79 ± 4.02	0.00 ± 0.00	1.01 ± 0.83	0.00 ± 0.00
MLP(5)	84.83 ± 6.04	14.50 ± 5.48	0.02 ± 0.02	0.58 ± 0.54	0.08 ± 0.11
MLP(5,5)	83.89 ± 6.29	15.55 ± 5.85	0.02 ± 0.02	0.52 ± 0.46	0.02 ± 0.02
RF	84.27 ± 6.50	14.24 ± 5.22	0.04 ± 0.06	1.45 ± 1.41	0.01 ± 0.02
GB	84.22 ± 5.94	14.63 ± 5.06	0.04 ± 0.08	1.09 ± 1.06	0.02 ± 0.03

Table H: EGA±sd test results averaged over patient for regressors with ADASYN oversampling.
